# Predictive factors of tinnitus after vestibular schwannoma surgery: a case-control study

**DOI:** 10.1186/s41016-024-00363-6

**Published:** 2024-04-03

**Authors:** Na You, Jiashu Zhang, Ding Zhang, Yue Zhao, Jun Zhang, Bainan Xu

**Affiliations:** 1 Medical School of Chinese PLA, Beijing, China; 2 Department of Neurosurgery, First Medical Center, Chinese PLA General Hospital, 28 Fuxing Road, Haidian District, Beijing, 100853 China

**Keywords:** Vestibular schwannoma, Tinnitus, Acoustic neuroma, Tinnitus Handicap Inventory

## Abstract

**Background:**

Tinnitus is very common in patients with vestibular schwannoma (VS). We analyzed the related factors of tinnitus after surgery.

**Methods:**

One hundred seventy-three patients diagnosed with unilateral VS operated via the retrosigmoid approach were included in the study. All patients underwent relevant examinations and completed the THI scale before surgery and 6 months after surgery. The prognosis of tinnitus was evaluated according to the changes in THI.

**Results:**

Of the 129 preoperative tinnitus patients, postoperative tinnitus resolved in 12.4%, improved in 29.5%, remained unchanged in 28.6%, and worsened in 29.5%. 18.2% of 44 patients without preoperative tinnitus appeared new-onset tinnitus postoperatively. Thirty-six patients never had tinnitus. Patients with smaller tumor sizes (≤ 3 cm) were more likely to experience preoperative tinnitus. Younger patients and those with serviceable hearing preoperatively were more likely to report their tinnitus unchanged or worsened. A new onset of postoperative tinnitus in the preoperative non-tinnitus group was found in better preoperative hearing function.

**Conclusions:**

In this study, 70% of patients had persistent tinnitus after vestibular schwannoma resection. The prognosis of tinnitus was influenced by age and preoperative hearing function. Tinnitus is a bothersome symptom and is often underestimated by doctors. Assessment of tinnitus is mandatory during the management of vestibular schwannoma.

## Background

Vestibular schwannoma (VS), also known as acoustic neuroma (AN), is a benign, slow-growing brain tumor arising from Schwann cells surrounding the vestibular nerve sheath [1, 2]. It represents the most common tumor of the cerebellopontine angle (CPA) and accounts for 80% of the CPA region [3]. Unilateral hearing loss and tinnitus are typical initial symptoms of vestibular schwannomas, accompanied by vertigo, gait ataxia, headache, and facial numbness.

VS-related tinnitus, a kind of subjective tinnitus, refers to the perception of noise in the absence of any external sound stimulation caused by vestibular schwannoma. Two-thirds of tinnitus persists. Tinnitus characteristics vary and are often described as a buzzing sound, a whistling sound, or other higher-pitched sound [4]. Unlike primary tinnitus, VS-related tinnitus is often characterized by aggravating when the external environment noise becomes louder [5], resulting in fear and avoidance behavior in noisy environments (such as roads, markets, and classrooms), which affects patients’ normal life and work. Approximately 80% of VS patients experience tinnitus preoperatively [6], of which 15.4% are severe or catastrophic tinnitus [7]. Moreover, tinnitus severity is closely related to mental health. Severe tinnitus can cause sleep disorders, affect residual hearing, and even lead to depression and anxiety, which makes the patient suffer a lot.

With the development of medical technology, the surgical prognosis of VS patients has been greatly improved. The goal of surgery has gradually transferred from tumor resection to improving quality of life with lower mortality and better neurological function preservation. However, most people focus on the preservation of facial nerve function and hearing, and tinnitus, which also has a great impact on the patient, is often ignored.

Although tinnitus relief is not the main goal of surgery, it is often one of the problems that patients hope to solve. Tinnitus is increasingly recognized as an important factor affecting a patient’s quality of life. It is necessary to fully discuss and inform patients about the occurrence and development of tinnitus and its potential impact on life before surgery. Therefore, this study aims to summarize the changes in tinnitus after vestibular schwannoma surgery and analyze the factors affecting the prognosis of VS-related tinnitus.

## Methods

In total, 173 patients diagnosed with unilateral VS were enrolled in this prospective study between August 2019 and February 2022 in the Department of Neurosurgery at the Chinese PLA General Hospital. Patients with recurrence, neurofibromatosis type 2 (NF2), bilateral tinnitus, and other otogenic diseases (such as Meniere’s disease and otitis media) were excluded. All the patients were treated with microsurgery via retrosigmoid approach by the same senior surgeon. The facial nerve and vestibulocochlear nerve were carefully identified and protected with the assistance of electrophysiological monitoring throughout the surgery. The main clinical manifestations were hearing loss, tinnitus, gait ataxia, and facial paralysis. One hundred twenty-nine (74.6%) patients had preoperative tinnitus and 44 (25.4%) had no tinnitus before surgery.

Preoperative clinical data, including gender, age, tumor side, preoperative hearing function, tumor size, internal auditory canal invasion, tumor consistency, and Tinnitus Handicap Inventory (THI), were collected. Surgical records were consulted to determine the degree of resection and cochlear nerve preservation during surgery. Forty-nine patients with tinnitus before surgery underwent comprehensive tinnitus examinations, including tinnitus type, tone frequency, and tinnitus loudness.

According to the American Academy of Otolaryngology-Head and Neck Surgery (AAO-HNS) Hearing Classification Scale [8], class A and B (PTA ≤ 50 dB, SDS ≥ 50%) are serviceable hearing and class C and D are non-serviceable hearing. Tumor size, the maximum diameter of the tumor in the cistern, and the tumor invasion in the internal auditory canal were measured on axial MRI. When the volume of the tumor cyst is between 1/3 and 2/3 of the total volume, it is recorded as a mixed type.

Tinnitus is a highly subjective feeling, and the distress caused by tinnitus is mainly reflected through various assessment scales. THI [9] is an effective self-reporting assessment for quantifying the impact of tinnitus on daily life. It consists of 25 items grouped into three subscales: functional, emotional, and catastrophic. Tinnitus severity was divided into five levels according to the scores: level 1 (mild 1–16 points), level 2 (light 18–36 points), level 3 (moderate 38–56 points), level 4 (severe 58–76 points), and level 5 (catastrophic 78–100 points) [10]. The higher the score, the more severe the tinnitus and the greater the impact on life.

After 6 months of follow-up, the changes in tinnitus were evaluated comprehensively according to the THI score before and after surgery and the subjective feelings of patients. Regardless of the preoperative THI, if there is no tinnitus after surgery (THI = 0), tinnitus is resolved. △THI (△THI=THI _post_- THI _pre_) ≤− 8 points, tinnitus improved. △THI = 0 points, tinnitus remained unchanged. △THI ≥ 8 points, tinnitus worsened. When the change in THI is within ± 8 points, the classification is based on the patient’s subjective feelings.

SPSS25.0 and GraphPad Prism 9 were used to perform statistical analysis and data plotting. Measurement data that conformed to the normal distribution were expressed as the mean (standard deviation). Measurement data that did not conform to the normal distribution were expressed as median (interquartile range, Q1–Q3) and analyzed using the Wilcoxon signed-rank test. Count data were expressed as frequencies and percentages, and analyzed using the chi-square test or Fisher’s exact test between different groups. The multivariate analysis was performed by binary logistic regression analysis. *P* < 0.05 were considered statistically significant.

## Results

**Fig. 1:**
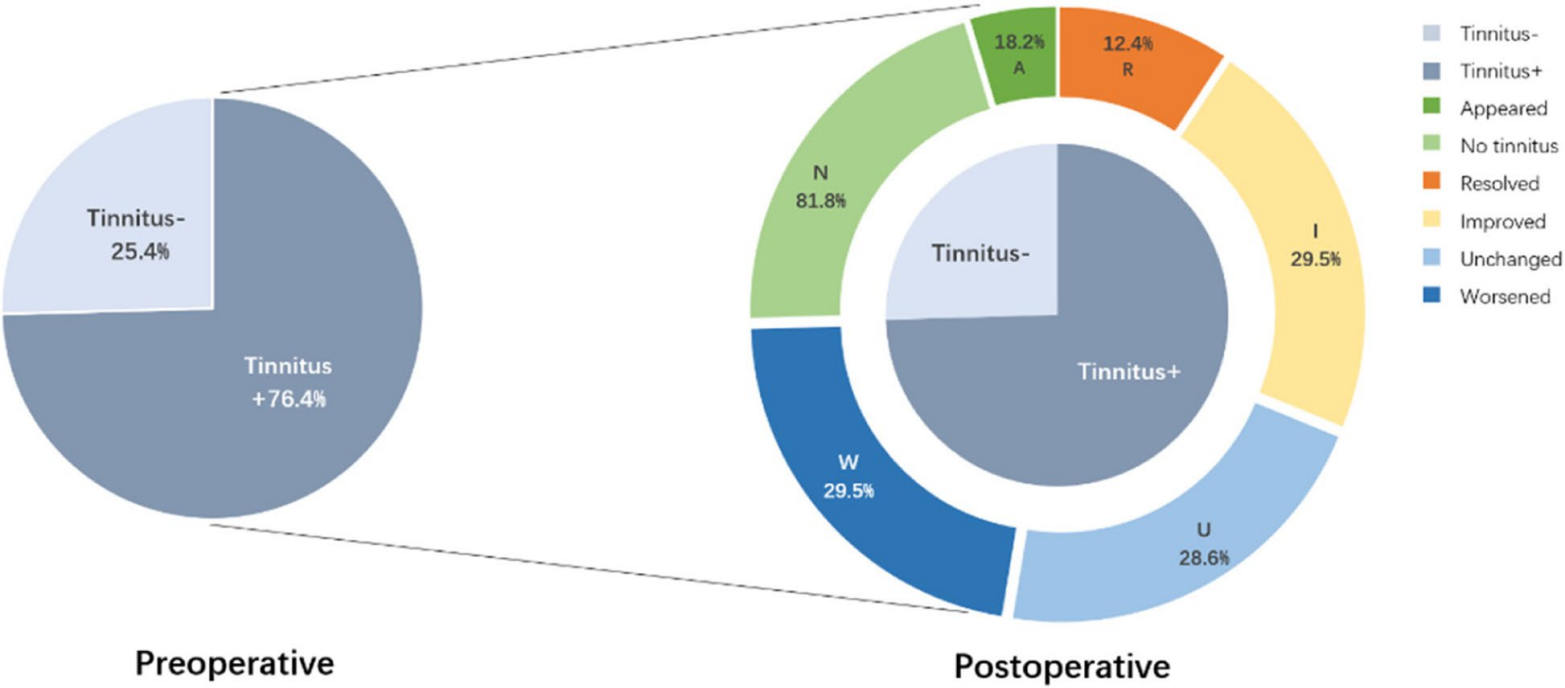
Changes of tinnitus in patients with vestibular schwannoma after surgery. Of the 173 patients, 129 patients had tinnitus and 44 patients had no tinnitus before surgery. After surgery, tinnitus resolved in 16 cases, improved in 38 cases, unchanged in 37 cases, worsened in 38 cases, and appeared in 8 cases, and 36 cases still had no tinnitus. Tinnitus+, patients with preoperative tinnitus; Tinnitus−, patients without preoperative tinnitus; R, resolved; I, improved; U, unchanged; W, worsened; N, no tinnitus; A, appeared

A total of 173 patients with unilateral VS were included in this study, including 74 males (42.8%) and 99 females (57.2%). The mean age was 47.6 ± 12.2 years old. The average duration of hearing loss was 26.5 months, and the average tumor size was 2.5 ± 1.1 cm. The remaining characteristics are shown in Table 1. Among the patients, 129 patients had preoperative tinnitus. After surgery, tinnitus resolved (R) in 16 cases (12.4%), improved (I) in 38 cases (29.5%), remained unchanged (U) in 37 cases (28.6%), and worsened (W) in 38 cases (29.5%). Forty-four patients had no tinnitus before surgery, 36 cases (81.8%) still had no tinnitus (N) after surgery, and 8 cases (18.2%) appeared tinnitus (A) (Fig. 1).

**Table 1:** Clinical characteristics of vestibular schwannoma

Characteristics	Tinnitus+	Tinnitus−	*P* value	*n* (%)
Gender				
Male	60	14	0.089	74 (42.8)
Female	69	30		99 (57.2)
Side				
Right	60	17	0.364	77 (44.5)
Left	69	27		96 (55.5)
Age			0.629	
> 50 years	62	23		85 (49.1)
≤ 50 years	67	21		88 (50.9)
Tumor size			0.008*	
≤ 3 cm	100	25		125 (72.3)
> 3 cm	29	19		48 (27.7)
ICA invasion			0.52	
≤ 1 cm	106	38		144 (83.2)
> 1 cm	23	6		29 (16.8)
Tumor consistency			0.085	
Solid	92	25		117 (67.6)
Cystic	9	2		11 (6.4)
Mixed	28	17		45 (26.0)
AAO-HNS classification			0.435	
Class A	24	13		37 (21.4)
Class B	26	7		33 (19.1)
Class C	21	2		23 (13.3)
Class D	58	22		80 (46.2)

*ICA* internal auditory canal, *AAO-HNS* American Academy of Otolaryngology–Head and Neck Surgery

*p < 0.05

### Microsurgery

All patients underwent tumor resection via the retrosigmoid approach. The incidence of preoperative and postoperative tinnitus was 74.6% and 69.9% (*c*^2^ = 0.923, *p* = 0.337), respectively. There was no statistical difference in tinnitus incidence before and after surgery. The average preoperative THI of patients with tinnitus was 24 (14, 40), and the postoperative mean THI was 20 (8, 38). A paired Wilcoxon signed-rank test was used to evaluate the preoperative and postoperative THI scores (*Z* = − 1.624 and *p* = 0.104). This indicated that tumor resection through the retrosigmoid approach would not increase the incidence of tinnitus in VS patients, nor would it exacerbate the severity of postoperative tinnitus.

### Preoperative tinnitus

The patients were divided into Tinnitus+ and Tinnitus− regarding their preoperative tinnitus status. The differences in factors such as gender, age, preoperative hearing function, tumor size, and internal auditory canal invasion were compared between the two groups. The results showed that patients with smaller tumor size (≤ 3 cm) were more likely to experience preoperative tinnitus than those with larger tumor size (> 3 cm) (*p* < 0.05). Other factors had no significant impact on the occurrence of VS-related tinnitus (Table 1).

### Postoperative tinnitus

**Fig. 2:**
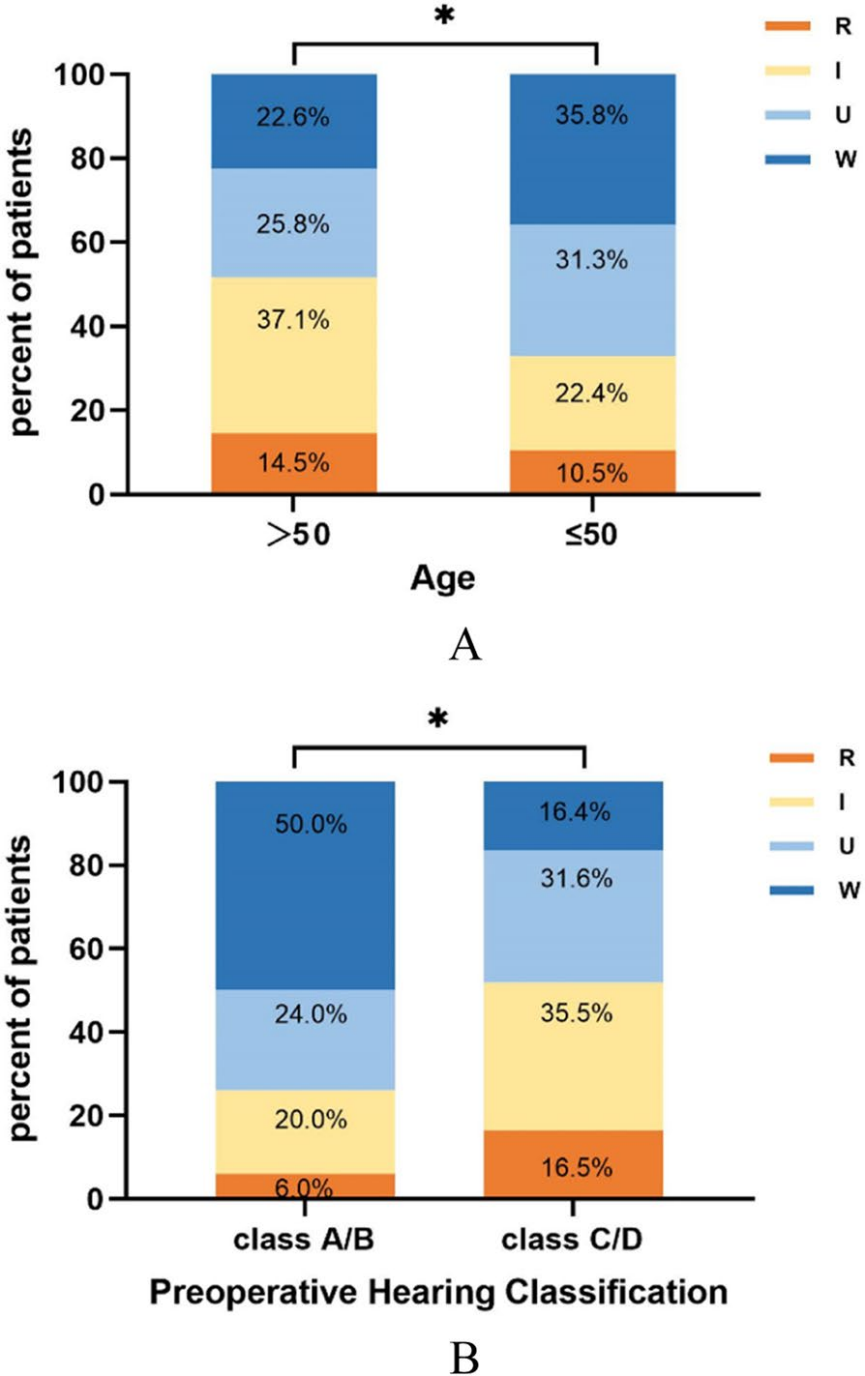
**A** Age and prognosis of tinnitus (*n* = 173). Patients > 50 years old were more likely to resolve or improve their postoperative tinnitus than patients ≤ 50 years old. **B** Preoperative hearing function and prognosis of tinnitus (*n* = 173). Patients with preoperative serviceable hearing were more likely to have unchanged or worsened tinnitus after surgery compared to those with non-serviceable hearing. *p < 0.05. R, resolved; I, improved; U, unchanged; W, worsened

**Fig. 3:**
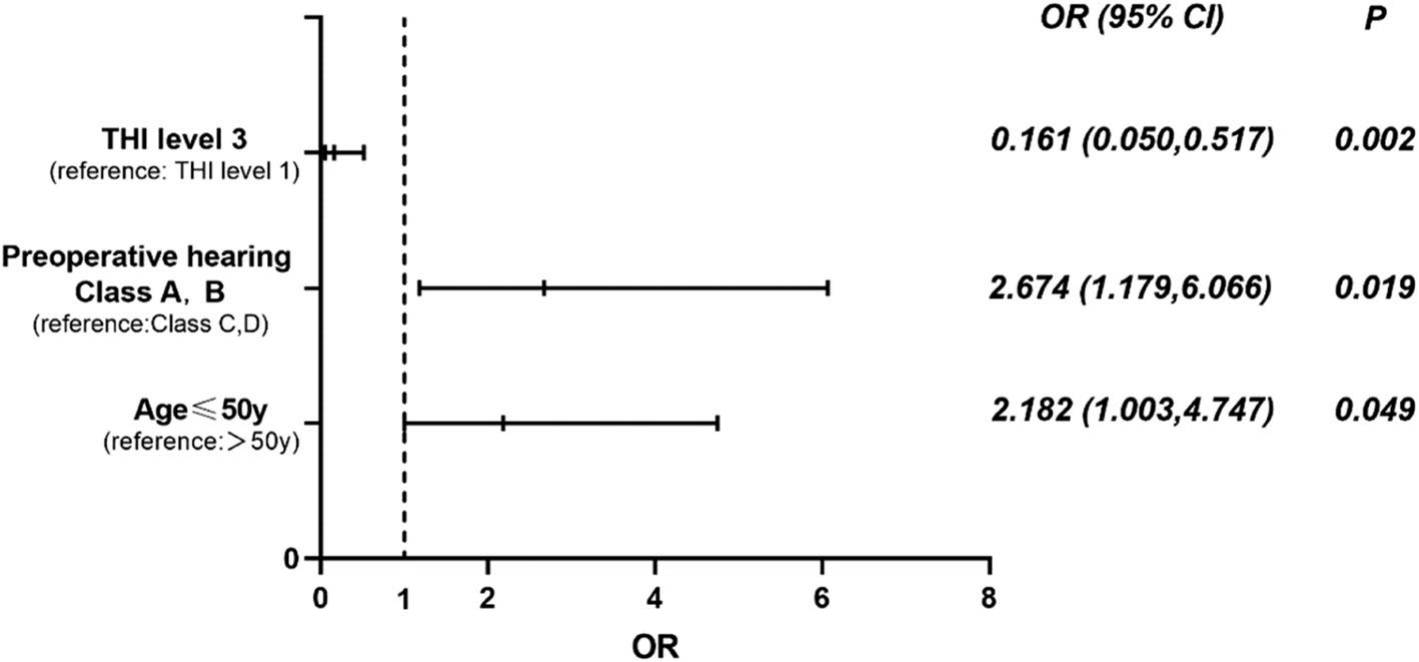
Binary logistic regression analysis. The worse prognosis of tinnitus in patients aged ≤ 50 years old was 2.182 times higher than patients > 50 years old, patients with preoperative serviceable hearing was 2.674 times higher than patients with non-serviceable hearing and in patients with preoperative THI level 3 was 0.161 times higher than those with THI level 1

According to the prognosis of tinnitus, the changes in postoperative tinnitus were divided into the R+I group and U+W group. The results showed that patients > 50 years old were more likely to report their tinnitus resolved or improved than patients ≤ 50 years old (*p* = 0.031, Fig. 2A). Patients with preoperative serviceable hearing were more likely to have unchanged or worsened tinnitus after surgery compared to those with non-serviceable hearing (*p* = 0.004, Fig. 2B). Patients with preoperative THI level 1 had the lowest postoperative tinnitus resolution and improvement rates, while other levels were all higher than level 1. However, only level 3 had a statistical difference from level 1 (*p* < 0.001). The results are shown in Table 2. Binary logistic regression analysis showed that age, preoperative hearing, and preoperative THI scale were the independent risk factors for the prognosis of tinnitus after surgery (Fig. 3).

**Table 2:** Influencing factors of postoperative tinnitus in vestibular schwannoma

	Changes of tinnitus		*c*^*2*^	*P* value
	R+I	U+W		
Gender (male/female)	29/25	31/44	1.931	0.165
Age (> 50 years/≤ 50 years)	32/22	30/45	4.665	0.031^*^
Preoperative hearing (Class A, B/Class C, D)	13/41	37/38	8.439	0.004^*^
Tumor size (≤ 3 cm/> 3 cm)	41/13	59/16	0.135	0.713
ICA invasion (≤ 1 cm/> 1 cm)	41/13	65/10	2.472	0.116
Tumor consistency (cystic/solid/mixed)	5/37/12	4/55/16	0.807	0.668
Cochlear nerve (+/−)	9/45	18/57	1.020	0.312
Degree of resection (total/subtotal)	46/8	62/13	0.146	0.702
THI			12.435	0.006^*^
Level 1	9	31		
Level 2	24	30		
Level 3	16	8	12.292	< 0.001^#^
Level 4、5	5	6		

*ICA* internal auditory canal, *R* resolved, *I* improved, *U* unchanged, *W* worsened

*p < 0.05

^#^Compared with level 1, *p* < 0.001

### Tinnitus frequency and loudness

**Fig. 4:**
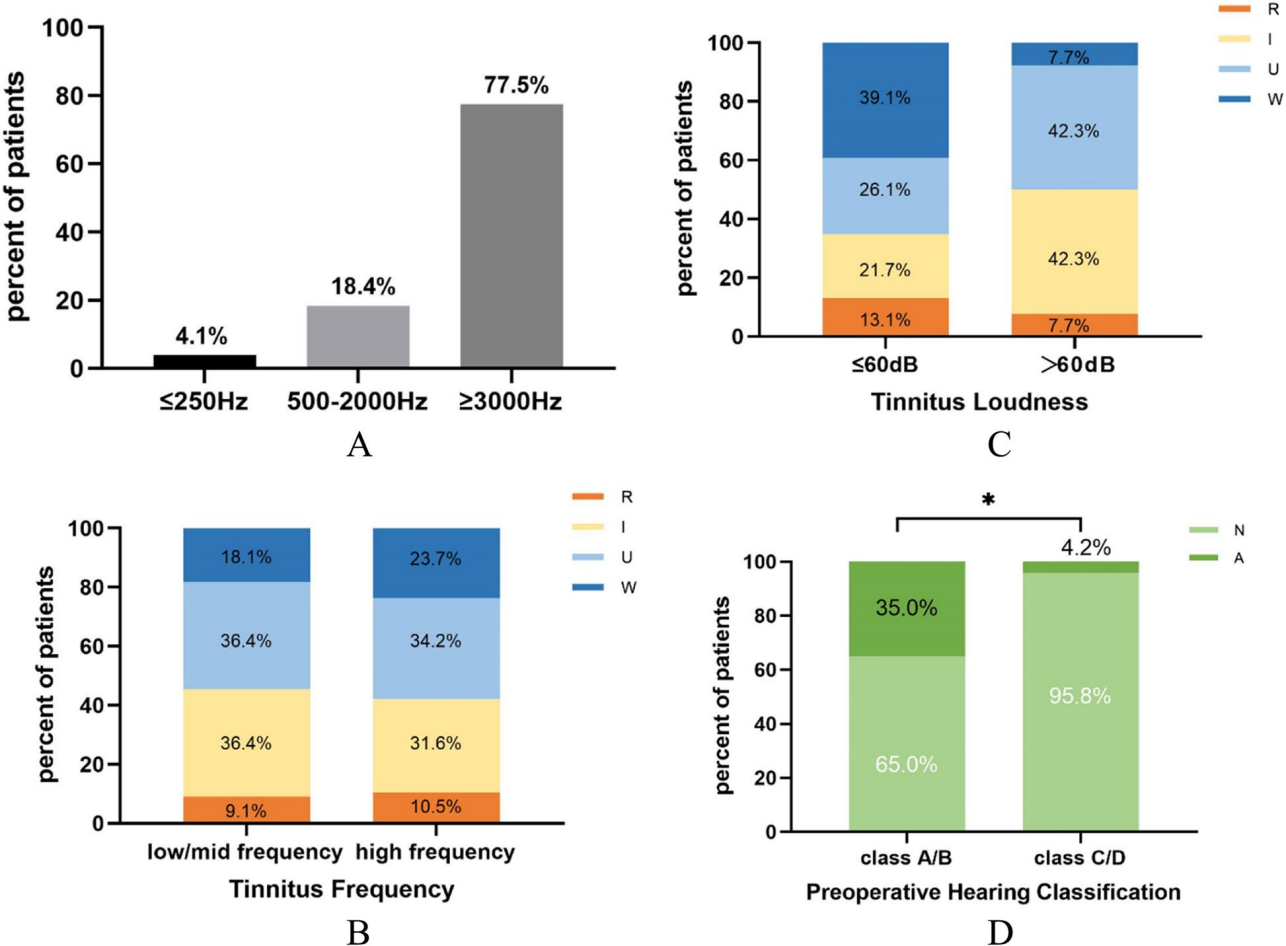
**A** Frequency distribution of preoperative tinnitus (*n* = 49). **B** Tinnitus frequency and prognosis of tinnitus (*n* = 49). **C** Tinnitus loudness and prognosis of tinnitus (*n* = 49). Frequency and loudness were not related to the prognosis of tinnitus. **D**: Preoperative hearing function and postoperative new-onset tinnitus (*n* = 44). Patients with preoperative serviceable hearing were more likely to experience new-onset tinnitus after surgery. *p < 0.05. R, resolved; I, improved; U, unchanged; W, worsened; N, no tinnitus; A, appeared

Forty-nine preoperative tinnitus patients completed comprehensive tinnitus examinations. 65.3% of tinnitus tones were pure tones, 34.7% were compound tones. The average frequency and loudness were 4 kHz and 61.5 ± 23.2 dB, respectively. Tinnitus frequency can be further characterized as low-frequency (≤ 250 Hz), mid-frequency (500–2000 Hz), and high-frequency (≥ 3000 Hz). There were 2, 9, and 38 cases in each group (Fig. 4A). Analyzing the impact of preoperative tinnitus frequency and loudness on postoperative tinnitus changes, it was found that there was no significant statistical difference in tinnitus frequency (*p* = 1.00) and loudness (*p* = 0.283) between R+I group and U+W group (Fig. 4B, C).

### Appeared tinnitus

Of the 44 patients without preoperative tinnitus, 8 cases had new-onset tinnitus after surgery, including 6 cases of THI level 1, 1 case of level 2, and 1 case of level 4. Patients with preoperative serviceable hearing (*p* = 0.015, Fisher’s exact test) were more likely to experience new-onset tinnitus after surgery (Fig. 4D). No significant difference was found between the no tinnitus (N) group and appeared tinnitus (A) group in terms of gender (*p* = 0.970), age (*p* = 0.126), tumor size (*p* = 1.000), internal auditory canal invasion (*p* = 0.297), cochlear nerve (*p* = 0.219), tumor consistency (*p* = 0.431), and degree of tumor resection (*p* = 1.000).

## Discussion

Tinnitus is the second most common symptom in patients with vestibular schwannoma besides hearing loss. Approximately 63–75% of VS patients complain about preoperative tinnitus, and about 10% of these patients have tinnitus as the presenting symptom [6]. Tinnitus often changes after surgery. Previous studies have found that about 15–66% of tinnitus resolved after surgery, 6.5–60% improved, 10–87% unchanged, and 6–50% worsened [6, 11–14], which is consistent with our study. Postoperative tinnitus troubles patients but is often ignored by us. Therefore, tinnitus treatment should be part of the comprehensive treatment of vestibular schwannoma. Changes in tinnitus should be estimated before surgery. With this information, it is possible for patients to better understand the potential impact of postoperative tinnitus before surgery, which will positively affect postoperative life quality.

Microsurgery via retrosigmoid approach is a standard treatment option for vestibular schwannoma. Both Gompel et al. [15] and Cao et al. [16] found that tinnitus improved after surgery regardless of whether a retrosigmoid approach, a translabyrinthine approach, or a middle cranial fossa approach was used. Kitamura et al. [17] found surgical approach did not affect the postoperative THI score. In our study, the incidence of tinnitus and THI score decreased after tumor resection via the retrosigmoid approach. Although there was no statistical difference compared with that preoperatively, to a certain extent, the retrosigmoid approach would not increase the occurrence of postoperative tinnitus or aggravate tinnitus. Moreover, patients with THI level 3 had a higher rate of tinnitus resolution and improvement after surgery, indicating that patients with moderate tinnitus benefit the most after surgery. It seems that the better the preoperative THI level, the worse the post-operative tinnitus prognosis. It should be noted that although tinnitus of some patients with THI level 1 and level 2 aggravates after surgery most of them still belong to mild and light tinnitus. Therefore, the change of postoperative tinnitus should not be the primary consideration in the decision of surgical treatment, and patients who meet the surgical indications are still recommended timely surgery.

Kohno et al. [12] pointed out that the prognosis of tinnitus in younger patients (< 45 years old) was worse than that in older patients (≥ 45 years old). Similarly, Bell et al. [13] also found that age was related to the prognosis of tinnitus. Patients under 50 years old were more likely to have tinnitus unchanged or worsened after surgery, which was consistent with our conclusion. Young patients generally have higher requirements for quality of life and bear greater life pressure because of the needs of occupation, social life and work. The poor prognosis of tinnitus in young patients may be related to their higher expectations and limited psychological acceptance. Postoperative psychotherapy and cognitive behavioral therapy should be part of the postoperative treatment for tinnitus patients with vestibular schwannoma, and the negative impact of tinnitus on life can be reduced by learning relevant processing skills.

The impact of tumor size on tinnitus is controversial. Some studies [18, 19] found preoperative tinnitus was not related to tumor size. While Kohno et al. [12] found the average tumor size in the preoperative tinnitus group was significantly smaller than that in the non-tinnitus group. In this study, patients with small tumors were more likely to develop preoperative tinnitus, and no statistically significant association was found between tumor size and postoperative tinnitus.

Some studies reported that patients with preoperative serviceable hearing had a worse prognosis for their tinnitus [6, 13, 18]. This study found preoperative hearing was related to the prognosis of tinnitus, but no relationship was found between cochlear nerve status and the prognosis of tinnitus. Preservation of the cochlear nerve is crucial to hearing preservation. A study in 367 patients reported that patients in whom the cochlear nerve was cut intraoperatively and the cochlear nerve was preserved as well as serviceable hearing had a better prognosis of tinnitus [12]. While the worst situation of tinnitus was cochlear nerve was preserved intraoperatively but the hearing was lost postoperatively. This indicates that cutting the cochlear nerve is effective in resolving or improving tinnitus. Incomplete damage to the cochlear nerve causes a poor prognosis for tinnitus, which may be due to the dysfunction of the cochlear nerve leading to the continuous reception of abnormal "sound signals" in the auditory cortex [20]. Therefore, regarding only the prognosis of tinnitus, it is recommended not to preserve the cochlear nerve when it is difficult to preserve hearing during surgery. On the other hand, preservation of cochlear nerve and hearing function can also improve tinnitus. Therefore, preservation of serviceable hearing should take precedence over solving tinnitus. With the development of medical technology, the preservation of the cochlear nerve provides a prerequisite for postoperative hearing reconstruction, and cochlear implantation will also have a positive impact on tinnitus [21]. Based on the above studies, we recommend that hearing preservation should be the principal objective for patients with serviceable preoperative hearing regardless of tumor size. If there is non-serviceable preoperative hearing or hearing cannot be preserved during surgery, cutting the cochlear nerve can be considered.

The tinnitus frequency of most VS patients is higher than 2000 Hz, mainly because the peripheral part of the eighth cranial nerve comes from the basal region of the cochlea and mainly transmits high-frequency sound signals. The tumor first compresses to the peripheral part of the nerve, affecting high-frequency hearing [22]. Wang et al. [10] found that patients with low-frequency and quieter preoperative tinnitus seemed to have a better postoperative prognosis than those with mid/high-frequency or louder preoperative tinnitus. We found that tinnitus frequency and loudness had no statistically significant association with postoperative tinnitus. This may be related to the limited number of patients who completed the tinnitus examination. Further researches is needed in this area.

Postoperative new-onset tinnitus is a special type of tinnitus that changes after surgery. Approximately, 22% of VS patients appeared tinnitus after surgery [12, 23], and in our study, it was 18.2%. Zhang et al. [23] found that patients with lower PTA had a higher incidence of new-onset tinnitus. We also found that patients with serviceable preoperative hearing were more likely to experience new-onset tinnitus after surgery. The THI score of new-onset tinnitus patients is mostly level 1, indicating that the new-onset tinnitus has little impact on the patients’ daily life. However, there were a small number of people who still could not tolerate the new symptoms after surgery. Therefore, new-onset tinnitus cannot be ignored. Adequate preoperative communication between patients and doctors is very necessary.

## Limitations

This study has several limitations. First, the assessment of tinnitus is subjective. Although THI can objectively assess tinnitus to a certain extent, objectively assessing the severity of tinnitus and its impact on quality of life remains a great challenge. Second, most of the patients did not complete the postoperative hearing examination and failed to evaluate their psychological status before surgery. Therefore, further research is needed on the relationship between postoperative hearing changes, psychological status, and tinnitus in VS patients. Third, this is a single-center study. The number of cases in some groups is small and the statistical efficiency is relatively limited. There may be some undiscovered differences between different groups. At the same time, the changes in postoperative hearing and tinnitus features may also be potential confounding factors. Therefore, we hope to cooperate with other hospitals in the future to conduct a multi-center, large-sample study.

## Conclusion

In summary, tinnitus often occurs in VS patients preoperatively, especially in patients with small tumors. The impact of tinnitus on VS patients is often underestimated. Surgery does not increase the incidence of tinnitus. A worse prognosis of postoperative tinnitus in the preoperative tinnitus group was found in younger patients and better preoperative hearing function. New-onset tinnitus is easier to occur in preoperative non-tinnitus patients with serviceable hearing. The future multicenter study and the study of the relationship between psychological state and the severity of tinnitus are valuable for promoting the understanding of tinnitus associated with vestibular schwannoma. For patients with postoperative persistent tinnitus, we can try medication, psychological intervention, and transcranial magnetic stimulation to help patients alleviate the problems caused by tinnitus.

## Data Availability

All data generated or analyzed during this study are included in this published article.

## Abbreviations

VS: Vestibular schwannoma
AN: Acoustic neuroma
CPA: Cerebellopontine angle
NF2: Neurofibromatosis type2
THI: Tinnitus Handicap Inventory
AAO-HNS: American Academy of Otolaryngology-Head and Neck Surgery
PTA: Pure-tone audiometry
